# Infectious, inflammatory, and clinical outcomes among patients prescribed trifluoperazine or amoxapine: a retrospective cohort study using real-world data

**DOI:** 10.3389/fmed.2026.1817814

**Published:** 2026-08-26

**Authors:** Omar Malik, Jorge Rodriguez-Fernandez, Aliu Opeyemi Yakubu, Wedad Farrag, Ernesto G. Miranda-Morales, Sara M. Dann, Ashok K. Chopra, Xiang Fang

**Affiliations:** 1 John Sealy School of Medicine, University of Texas Medical Branch at Galveston, Galveston, TX, United States; 2 Department of Neurology, University of Texas Medical Branch at Galveston, Galveston, TX, United States; 3 School of Veterinary Medicine, Louisiana State University, Baton Rouge, LA, United States; 4 Department of Internal Medicine, University of Texas Medical Branch at Galveston, Galveston, TX, United States; 5 Department of Microbiology and Immunology, University of Texas Medical Branch at Galveston, Galveston, TX, United States; 6 Moody Brain Health Institute, University of Texas Medical Branch at Galveston, Galveston, TX, United States

**Keywords:** amoxapine, drug repurposing, infectious disease, psychotropic drugs, retrospective cohort, trifluoperazine, TriNetX

## Abstract

**Introduction:**

The rise of antimicrobial resistance and limited development of new antibiotics have increased interest in drug repurposing. Preclinical evidence suggests that certain psychotropic drugs, including trifluoperazine (TFP) and amoxapine (AXPN), possess antimicrobial or immunomodulatory properties. However, their effects on infection risk in clinical populations are largely unknown. This study evaluated whether TFP or AXPN use is associated with altered risks of infectious illnesses, systemic inflammation, and mortality compared to patients not taking antidepressants.

**Methods:**

This retrospective cohort study utilized TriNetX, a federated network of de-identified electronic health records from over 75 million patients. Patients prescribed TFP, AXPN, fluoxetine, or no antidepressants/antipsychotics (general control) were assigned to mutually exclusive cohorts. Age- and sex-matched comparisons were conducted for four groups. Assessed outcomes included bacterial and viral infections (e.g., *Clostridioides difficile*), severe acute respiratory syndrome coronavirus 2, an etiological agent of COVID-19, as well as for pneumonia and sepsis, inflammatory markers (C-Reactive Protein, Erythrocyte Sedimentation Rate, ferritin, and procalcitonin), Intensive Care Unit admission, and mortality. Risk ratios (RR), odds ratios (OR), 95% confidence intervals, and Kaplan–Meier survival analyses were performed for significant findings.

**Results:**

Overall, 2,177 TFP and 834 AXPN patients met the inclusion criteria. TFP was associated with significantly lower rates of ENT/dental infections across all three age groups compared with general controls (RR 0.316–0.405, all *p* ≤ 0.002). It was also consistently associated with increased leukocytosis across all age strata. Reduced COVID-19 incidence was observed primarily in older adults (70–90 years) versus general controls. AXPN showed fewer associations overall, with a significant reduction in *C. difficile* infection (RR 0.458, *p* = 0.025) and lower leukocytosis (RR 0.685, *p* = 0.012) in the 70–90 age group compared with fluoxetine. Most other outcomes, including pneumonia, sepsis, and other inflammatory markers, did not differ significantly when compared to general control or fluoxetine groups.

**Discussion:**

These exploratory findings identify potential signals linking TFP and AXPN to infection susceptibility. Given the study design, these observations are hypothesis-generating and require confirmation in future prospective studies.

## 1 Introduction

The global burden of infectious diseases has been compounded by the accelerating crisis of antimicrobial resistance (AMR) emergence in nosocomial and community-acquired bacterial pathogens (1). This worldwide predicament has not only threatened the effectiveness of existing antibiotics, but the result of overall higher mortality rates and healthcare costs is totally unprecedented (2). Overuse and misuse of antibiotics, both medically and across agricultural sectors, have posed excessive selective pressures, enabling resistant microbial populations to proliferate. At the same time, there has been a slowing of new antimicrobial compound research and development due to high expenses, lengthy regulatory timelines for approval, and poor success rates for conventional drug development routes (2). These challenges underscore the urgent need for new approaches to infectious disease management, especially by repurposing available Food and Drug Administration (FDA)-approved drugs with known safety profiles towards their new antimicrobial or immunomodulatory roles.

One such promising approach is host-directed therapy, which leverages host-specific mechanisms of immunity as opposed to engaging the pathogen directly (3). This strategy reduces selective pressure applied to microbial populations and can potentially slow the emergence of AMR (4). Through an initial high-throughput screen of 780 FDA-approved compounds, Andersson et al. (5) identified 94 non-antibiotic drugs that protected murine macrophages from *Yersinia pestis*-induced cytotoxicity. Trifluoperazine (TFP) and amoxapine (AXPN), which are typical antipsychotic and antidepressant drugs, respectively, were among the most effective tested compounds. Though neither drug exhibited antibacterial activity, both significantly improved survival in murine models of pneumonic plague, suggesting a host-modulating mechanism.

Subsequent studies have demonstrated that TFP and AXPN offered protective benefits against several other infectious diseases in animal models (5, 6). For example, it was found that both drugs significantly improved survival in mice infected with *Clostridioides difficile (C. difficile)* and *Salmonella enterica* as a standalone therapy, and particularly, when combined with standard antibiotics like vancomycin or levofloxacin at sub-therapeutic doses (5, 6). These results suggest an additive or synergistic effect, where non-antibiotic psychotropic medications enhance host immune responses to clear infection, independent of their direct impact on directly impacting bacterial load. Follow-up work in 2020 revealed that AXPN, especially when administered alongside TFP or doxapram, a breathing stimulant, activates key innate immune pathways, including those involving IL-22, IL-33, production of host antimicrobial production, and neutrophil recruitment—and loses efficacy when these immune pathways are disrupted (4).

Complementary findings in human populations support the notion that centrally acting drugs may influence infectious outcomes (7–9). A retrospective study by Oskotsky et al. reported reduced COVID-19 mortality among patients prescribed selective serotonin reuptake inhibitors (SSRIs) like fluoxetine or fluvoxamine, an effect attributed to the immunomodulatory and anti-inflammatory properties of these agents (7). More recent mechanistic work has shown that fluoxetine enhances survival in sepsis cases by increasing interleukin 10 (IL-10), which mitigates metabolic stress and cardiac dysfunction during systemic infection (8). This immunometabolism defense highlights the broader off-target benefits of SSRIs in infectious settings. These findings also align with emerging models of host defense that distinguish between resistance—eliminating pathogens—and tolerance—limiting the damage caused by infection (9). In this light, psychotropic medications may enhance health not by directly attacking pathogens, but by supportive adaptive physiological processes that maintain host resilience. This shift toward studying the biology of health, rather than solely disease, provides a novel framework for exploring the therapeutic potential of these widely prescribed agents.

Despite promising preclinical and clinical findings, there is a lack of real-world evidence examining the potential anti-infective or immunomodulating effects of TFP and AXPN in larger human populations. We hypothesized that prior exposure to TFP or AXPN would be associated with reduced risk of common community infections and critical clinical events, such as ICU admission or mortality. Given their distinct pharmacological actions, in combination with their current use in psychiatry, TFP and AXPN offer a unique area of study for determining if their benefits translate beyond mental health treatment. Using TriNetX, we performed age- and sex-matched comparisons and included an active-comparator analysis with fluoxetine to determine whether observed associations persisted across age strata. Fluoxetine was prespecified as the active comparator given its reported immunomodulatory effects in infectious contexts (7, 8), enabling us to evaluate TFP or AXPN against a biologically plausible alternative rather than solely against non-exposed controls (general controls).

## 2 Methods

### 2.1 Study design

We conducted a retrospective, observational cohort study using the TriNetX federated Electronic Health Record network to determine if prior treatment with TFP or AXPN was associated with different levels of risk related to several infectious, inflammatory, and clinical events. We focused on a broad set of clinically significant endpoints, including *C. difficile* infection, pneumonia, sepsis, COVID-19, tuberculosis (TB), herpes simplex virus infections, bacteremia, bronchitis/lower respiratory tract infections (LRTIs), urinary tract infections (UTIs), ear/nose/throat (ENT)/dental infections, neutrophilia, elevated levels of C-reactive protein (CRP), elevated erythrocyte sedimentation rate (ESR), elevated ferritin, elevated procalcitonin, ICU admissions, and mortality. To minimize confounding from possible psychiatric disorders or demographic differences, each drug treatment cohort group was compared to an age and sex-matched group of patients without baseline antidepressant and antipsychotics exposure, selected using the general exam diagnosis code Z00.00.

As a secondary analysis, we also compared TFP and AXPN to an age and sex-matched cohort of patients prescribed fluoxetine, an SSRI, with evidence of immunomodulatory benefits in infectious contexts, as discussed earlier (7, 8). Additional measures were taken to reduce confounding: all analyses were adjusted for the Charlson Comorbidity Index, and results were stratified by age group to control for age-related variability in infection and immune response.

### 2.2 Criteria for inclusion and exclusion

Participants eligible for inclusion in the study were aged 18 and above with at least one recorded prescription for either TFP or AXPN from inception to October 2025. Patients prescribed both TFP and AXPN were excluded to ensure mutually exclusive cohorts. A large match cohort was selected as a comparison group, which consisted of patients without exposure to TFP or AXPN in the network who underwent a physical exam, denoted by ICD-10 code Z00.00 and those who were at least 18 years old (general control). A similar process was used to create a fluoxetine control cohort for secondary analysis. Four overall comparisons were examined: AXPN vs. general control, TFP vs. general control, AXPN vs. fluoxetine, and TFP vs. fluoxetine. To ensure the validity of the infectious outcome assessments, only newly documented diagnoses of cases developed after the drug exposure starting date were counted. Exclusion criteria were the presence of cancer, severe immunosuppression, and unspecified psychosis that might confound the risk for infection or influence immune status, though this may limit generalizability. These exclusion criteria were applied to all treatment and control groups to reduce residual confounding due to underlying chronic systemic illness and to enhance the comparability between treatment and control groups.

### 2.3 Data collection

The data used in this study were drawn solely from the TriNetX platform, which aggregates structural clinical data covering demographic information, medication history, diagnoses (using ICD-10 codes), laboratory results, and clinical outcomes from a variety of healthcare organizations. For the infectious outcomes analysis, the primary exposures were defined by the recorded prescription of TFP or AXPN, with the first date of documented prescription being the index date. Patients were followed for 2 years from the index date for outcome assessment. Infectious outcomes were identified using validated ICD-10 codes within this period: *A04.7, B96.89, K52.9,* and *K52.89* for *C. difficile*, *U07.1* for COVID-19, codes *J12-J18* for pneumonia, *A41* for sepsis., *A15-A19* for TB, *B00* for herpes simplex virus, *R78.81* for bacteremia, *J20-J22* for bronchitis and LRTIs, *N39.0* for UTIs, and *K04.7, H60,* and *J02* for ENT/dental infections. Clinical events, including ICU admissions and mortality, were also assessed within the same two-year period. The two-year window for infectious clinical outcomes was selected to balance between sufficient outcome capture and minimizing unrelated long-term events. This timeframe is also temporally plausible given that antipsychotic and antidepressant prescriptions are often continued for extended periods in clinical practice, allowing sufficient time for any potential immunologic or infectious effects to emerge (10).

Inflammatory markers were also evaluated. The first was laboratory-confirmed neutrophilia, an elevated absolute neutrophil count above the institutional reference range, 8,000 neutrophils per microliter of blood (11). The second inflammatory marker outcome was elevated CRP levels, defined as greater than 10 milligrams per deciliter of blood (12). Additional markers included elevated erythrocyte sedimentation rate (ESR), elevated ferritin, and elevated procalcitonin, all of which were assessed based on whether values exceeded institutional upper reference limits, which were 20 mm/h, 330 ng/mL, and 0.10 ng/mL, respectively (12). To ensure temporal relevance to medication exposure, laboratory values were only included if recorded within 2 years following the index prescription, reflecting a biologically plausible window for drug-related inflammatory modulation.

### 2.4 Data analysis

Descriptive statistics were calculated to summarize the demographic and clinical characteristics of the patients in different cohorts. To evaluate the strength of the observed associations, risk ratios (RR) and their respective 95% confidence intervals were calculated. For the statistically significant outcomes in the primary analysis, additional characterization was performed through Kaplan–Meier survival analysis to estimate event-free survival over time. Log-rank tests enabled the detection of differences in the survival curves, and hazard ratios (HR) were calculated to quantify the relative risk of infection onset. All analyses were conducted separately for the three stratified age groups (18–49, 50–69, and 70–90 years) to evaluate whether associations differed across stages of adulthood characterized by distinct infection risks, comorbidity burdens, and immune system function. These groups were selected to represent younger, middle-aged, and older adult populations while maintaining adequate sample sizes and event rates for statistical analysis, allowing assessment of age-related differences in host immune responses and susceptibility to infectious outcomes. Sensitivity analyses were performed to assess the robustness of the primary findings using shorter follow-up periods of 6 and 12 months after the index prescription date. Additionally, a direct head-to-head comparison between the TFP and AXPN cohorts was conducted using age-, sex-, and Charlson Comorbidity Index-matched groups to evaluate potential differences in outcomes between the two drugs. Given the large number of outcomes examined, we acknowledge the increased risk of type I error. No correction for multiple comparisons was applied, as this study was designed as an exploratory, hypothesis-generating analysis rather than a confirmatory one. Accordingly, all *p*-values are reported as nominal, and statistical significance should be interpreted with caution, particularly for results near the conventional threshold (*p* ≈ 0.05).

## 3 Results

### 3.1 Characteristics of the cohort

A total of 2,177 patients prescribed TFP and 834 patients prescribed AXPN met the inclusion criteria and were included in the study. These patients were age- and sex-matched to general controls (no baseline antidepressant or antipsychotic exposure) and to fluoxetine-treated controls (Table 1). The TFP and AXPN groups had higher mean ages (65 and 62 years, respectively) than both the general control (52 years) and fluoxetine (50 years) groups. Women comprised the majority in all groups, although the proportions varied: 62.6% in the TFP group, 61.5% in the AXPN group, compared to 69.8% in the fluoxetine group and 53.6% in the general control group. Racial distribution also varied, with the AXPN and fluoxetine groups having the highest proportions of White patients (67.7 and 73.1%, respectively) and the TFP group showing the highest proportion of Asian patients (10.1%). Both TFP and AXPN groups had similar proportions of African American patients (10.7 and 12.1%, respectively), closely aligned with the general control population (10.6%) but higher than in the fluoxetine group (8.2%).

**Table 1:** Demographic characteristics of TFP, AXPN, general control (without baseline antidepressant or antipsychotic exposure), and fluoxetine control groups of patients.

Demographic characteristics	TFP group (*n* = 2,177)	AXPN group (*n* = 834)	General control group (*n* = 15,480,659)	Fluoxetine group (*n* = 1,730,339)
Age				
Mean Age	65	62	52	50
Gender				
% Women	62.6	61.5	53.6	69.8
% Men	37.4	38.5	46.4	30.2
Race				
% White	54.3	67.7	55.7	73.1
% African American	10.7	12.1	10.6	8.2
% Asian	10.1	1.2	5.1	1.8
% American Indian	0.5	1.2	0.3	0.4
% Native Hawaiian	0.5	0.0	0.6	0.2
% Other/ Unknown race	24.5	18.7	27.8	16.4
% Major depressive disorder	9.2%	11.9%	2.5%	19.3%
% Generalized anxiety disorder	12.2%	11.9%	5.3%	20.3%
% Bipolar disorder	15.8%	7.6%	0.6%	6.2%
% Schizophrenia	29.6%	4.3%	0.6%	1.3%
% Diabetes mellitus	21.7%	25,5%	4.4%	14.6%
Cardiovascular disease	60.7%	58.2%	17.4%	45.0%
Chronic pulmonary disease	22.3%	25.0%	10.5%	22.2%
Chronic kidney disease	11.0%	12.3%	0.9%	6.8%

Following propensity score matching on age and sex, the TFP cohort was compared with 1,994 general controls and 1,985 fluoxetine controls. The AXPN cohort was compared with 829 general controls and 828 fluoxetine controls (Table 2). Mean ages across all matched groups were similar, ranging from 54.5 to 55.2 years. Gender distribution was also comparable, with women comprising between 62.2 and 63.7% of each matched cohort. Men accounted for approximately 36.3 to 37.8% across groups.

**Table 2:** Post-propensity score matching demographic characteristics of TFP, AXPN, general control (without baseline antidepressant or antipsychotic exposure), and fluoxetine control groups of patients.

Demographic characteristics	General control matched to TFP (*n* = 1,994)	General control matched to AXPN (*n* = 829)	Fluoxetine control matched to TFP (*n* = 1,985)	Fluoxetine control matched to AXPN (*n* = 828)
Age				
Mean Age	55.2	54.5	55.1	54.5
Gender				
% Women	62.2	62.6	63.7	62.3
% Men	37.8	37.4	36.3	37.7
% Diabetes	17.5	19.3	19.9	21.1
% Cardiovascular disease	44.6	45.0	50.2	50.0
% Chronic pulmonary disease	19.8	19.5	25.4	26.2
% Chronic kidney disease	7.3	7.3	9.3	8.9

### 3.2 Trifluoperazine (TFP) outcomes

In this stratified analysis, we evaluated the association between TFP use and various infectious and inflammatory outcomes in comparison to age-, sex-, and Charlson comorbidity index-matched general controls across three age groups: 70–90, 50–69, and 18–49 years.

As shown in Table 3, TFP was consistently associated with reduced rates of ENT/dental infections in all three age groups, reaching statistical significance across the board (RR: 0.316 in age group 70–90 [*p =* 0.002], 0.395 in age group 50–69 [*p =* 0.001], and 0.405 in age group 18–49 [*p =* 0.0002]). Similarly, bronchitis/LRTIs were significantly reduced in the oldest cohort (RR: 0.400, *p* = 0.002), but not noted in younger age groups.

**Table 3:** Association between TFP use and infectious, inflammatory, and clinical outcomes by age group compared to general control (without baseline antidepressant or antipsychotic exposure) patients.

Outcomes	Age 70–90 (*n* = 871)		Age 50–69 (*n* = 668)		Aged 18–49 (*n* = 399)	
	Risk ratio	*p*-value	Risk ratio	*p*-value	Risk ratio	*p*-value
*C. diff*	1.100 (0.68,1.79)	0.700	1.333 (0.64,2.80)	0.450	1.900 (0.90,4.04)	0.089
Pneumonia	1.122 (0.76,1.68)	0.544	1.900 (0.89,4.06)	0.091	1.000 (0.42,2.38)	1.000
Sepsis	1.474 (0.83,2.62)	0.183	1.200 (0.52,2.76)	0.667	1.000 (0.42,2.38)	1.000
COVID-19	0.646 (0.42,1.00)	0.050*	1.000 (0.42,2.39)	1.000	1.000 (0.42,2.38)	1.000
TB	-	-	-	-	-	-
HSV	1.000 (0.42,2.39)	1.000	1.000 (0.42,2.39)	1.000	1.000 (0.42,2.39)	1.000
Bacteremia	0.909 (0.39,2.13)	0.826	1.000 (0.42,2.39)	1.000	1.200 (0.52,2.76)	0.670
Bronchitis/ LRTIs	0.400 (0.22,0.74)	0.002*	0.769 (0.34,1.74)	0.528	1.000 (0.42,2.39)	1.000
UTIs	1.048 (0.75,1.47)	0.782	0.581 (0.33,1.03)	0.058	1.214 (0.60,2.44)	0.585
ENT/Dental infections	0.316 (0.17,0.60)	0.0002*	0.395 (0.22,0.71)	0.001*	0.405 (0.23,0.73)	0.002*
Inc. CRP	1.269 (0.77,2.10)	0.354	1.000 (0.42,2.39)	1.000	1.000 (0.42,2.38)	1.000
Inc. ESR	0.826 (0.45,1.51)	0.532	1.400 (0.63,3.13)	0.410	–	–
Inc. leukocytes	1.778 (1.40,2.26)	<0.0001*	1.744 (1.20,2.54)	0.003*	3.933 (2.68,5.77)	<0.0001*
Mortality	2.000 (0.94,4.25)	0.065	1.200 (0.52,2.76)	0.667	–	–
ICU admission	1.000 (0.42,2.39)	1.000	1.000 (0.42,2.39)	1.000	–	–

*p-values less than 0.05 are marked with an asterisk.

An increase in risk of leukocytosis was observed among TFP users. The risk of elevated leukocyte counts was significantly higher in all groups, with the highest risk seen in younger adults (RR: 3.933 in age group 18–49; RR: 1.744 in age group 50–69; RR:1.778 in age group 70–90; with *p* values ranging from 0.003 to < 0.0001). There was a statistically significant reduction in COVID-19 risk associated with TFP use in the age 70–90 cohort (RR: 0.646, *p* = 0.050), while no difference was seen in younger groups. Other infectious outcomes such as pneumonia, *C. difficile*, and sepsis did not show statistically significant differences in any cohort. The mortality risk seemed somewhat higher among the oldest cohort (RR: 2.000, *p* = 0.065), though not statistically significant, and was not observed in younger groups. Therefore, the data should be interpreted cautiously. ICU admission did not differ between TFP users and controls in any group (Table 3).

When comparing TFP-exposed patients to those prescribed fluoxetine, several distinct trends emerged across age groups in terms of infectious, inflammatory, and clinical outcomes (Table 4). In the older adult group (ages 70–90), TFP use significantly lowered the risk of COVID-19 compared to fluoxetine (RR = 0.443, 95% CI: 0.30–0.65, *p* < 0.0001). However, no other infectious or inflammatory outcomes reached statistical significance in this age group. TFP use in this cohort of older adults was associated with elevated leukocytosis (RR = 1.468, 95% CI: 1.17–1.84, *p* = 0.007). Age 50–69 years showed a significantly reduced risk of ENT/dental infections compared to fluoxetine (RR = 0.441, 95% CI: 0.24–0.80, *p* = 0.006). Other outcomes—including sepsis, pneumonia, and inflammatory markers—did not differ meaningfully between groups.

**Table 4:** Association between TFP use and infectious, inflammatory, and clinical outcomes by age group compared to patients taking fluoxetine.

Outcomes	Age 70–90 (*n* = 845)		Age 50–69 (*n* = 605)		Aged 18–49 (*n* = 620)	
	Risk ratio	*p*-value	Risk ratio	*p*-value	Risk ratio	*p*-value
*C. diff*	0.861 (0.54,1.38)	0.533	0.938 (0.47,1.88)	0.856	1.000 (0.44,2.26)	1.000
Pneumonia	1.325 (0.89,1.98)	0.166	1.000 (0.54,1.87)	1.000	1.000 (0.44,2.26)	1.000
Sepsis	1.182 (0.68,2.07)	0.558	1.091 (0.49,2.45)	0.833	1.000 (0.44,2.26)	1.000
COVID-19	0.443 (0.30,0.65)	< 0.0001*	0.692 (0.46,1.04)	0.077	0.548 (0.37,0.81)	0.003*
TB	0.825 (0.53,1.30)	0.402	1.000 (0.42,2.39)	1.000	-	-
HSV	1.000 (0.42,2.39)	1.000	1.000 (0.42,2.39)	1.000	1.000 (0.42,2.36)	1.000
Bacteremia	1.000 (0.42,2.39)	1.000	-	-	1.000 (0.42,2.36)	1.000
Bronchitis/ LRTIs	0.778 (0.39,1.55)	0.475	0.714 (0.32,1.60)	0.410	1.000 (0.42,2.36)	1.000
UTIs	1.016 (0.72,1.43)	0.925	0.913 (0.51,1.63)	0.759	1.000 (0.42,2.36)	1.000
ENT/Dental infections	0.522 (0.26,1.04)	0.060	0.441 (0.24,0.80)	0.006*	1.182 (0.54,2.59)	0.645
Inc. CRP	0.825 (0.53,1.30)	0.402	0.818 (0.44,1.51)	0.520	1.000 (0.42,2.36)	1.000
Inc. ESR	0.594 (0.34,1.04)	0.065	1.000 (0.42,2.39)	1.000	–	–
Inc. leukocytes	1.468 (1.17,1.84)	0.007*	1.15 (0.83,1.60)	0.402	1.500 (0.74,3.05)	0.259
Mortality	1.235 (0.66,2.33)	0.512	1.000 (0.42,2.39)	1.000	–	–
ICU admissions	1.000 (0.42,2.39)	1.000	–	–	–	–

*P*-values less than 0.05 are marked with an asterisk.

Kaplan–Meier survival curves revealed significantly improved COVID-19-free survival among older adults (ages 70–90) prescribed TFP, compared to both general controls and fluoxetine users (Table 5). When compared to general controls, the survival probability for COVID-19 infection in the TFP group was 93.7% (*p* = 0.027), corresponding to a hazard ratio (HR) of 0.626, indicating a 37.4% reduced hazard of COVID-19 infection. The effect was even more pronounced when comparing TFP to fluoxetine users in the same age group (survival = 94.3%, *p* = 0.003, HR = 0.552). This protective effect against COVID-19 was also observed in young adults (18–49); when comparing TFP to fluoxetine (survival = 93.5%, *p* = 0.0004, HR = 0.483). TFP was associated with significantly improved ENT/dental infection-free survival across all age groups compared to general controls. Among older adults (70–90), the TFP group had a survival probability of 98.2% (*p* = 0.015, HR = 0.417), while the middle-aged (50–69) and younger adults (18–49) showed similarly favorable outcomes (survival = 96.6 and 95.9%; *p* = 0.003 and 0.007; HR = 0.421 and 0.478, respectively). Notably, when compared to fluoxetine, this benefit persisted in the 18–49 age group (*p* = 0.003, HR = 0.446), while the difference in the 50–69 group was not significant (*p* = 0.165).

**Table 5:** Kaplan–Meier survival analysis for statistically significant infectious and laboratory outcomes in patients by age-stratified group comparisons of TFP vs. control and fluoxetine.

Outcome	Group (Age range)	Survival probability	Log rank *p*-value	Hazard ratio	Proportionality *p*-value
COVID-19	T vs. C (70–90)	93.737	0.0270	0.626	0.626
	T vs. F (70–90)	94.265	0.003	0.552	0.785
	T vs. F (18–49)	93.464	0.0004	0.483	0.145
Bronchitis/ LRTIs	T vs. C (70–90)	97.997	0.066	0.541	0.307
ENT/Dental infections	T vs. C (70–90)	98.213	0.015	0.417	0.608
	T vs. C (50–69)	96.582	0.003	0.421	0.484
	T vs. C (18–49)	95.923	0.007	0.478	0.920
	T vs. F (50–69)	96.142	0.165	0.669	0.174
	T vs. F (18–49)	95.923	0.003	0.446	0.443
Leukocytosis	T vs. C (70–90)	78.524	<0.0001	2.198	0.092
	T vs. C (50–69)	87.514	0.011	1.638	0.095
	T vs. C (18–49)	76.656	<0.0001	4.721	<0.0001
	T vs. F (70–90)	79.281	0.125	1.202	0.060
	T vs. F (18–49)	78.775	<0.0001	2.417	0.004

T = TFP; C = General Control; F = Fluoxetine. P-values less than 0.05 are considered significant.

Among older adults, TFP demonstrated a trend toward improved survival in bronchitis/LRTIs compared to general controls (survival = 98.0%, *p* = 0.066, HR = 0.541), though this result did not reach statistical significance (Table 5). No survival differences for this outcome were observed in other groups or comparisons. An increase in leukocytosis-related event incidence was observed among TFP users across all age groups when compared to general controls. The effect was strongest in the youngest cohort (18–49), where the survival probability was lowest at 76.6% and HR was highest at 4.721 (*p* < 0.0001), with significant proportional hazards violation (*p* < 0.0001), suggesting non-proportional risk over time. Older adults (70–90) also experienced a significantly increased hazard of leukocytosis (HR = 2.198, *p* < 0.0001) when compared to a general control group, but not in older adults compared to fluoxetine users (HR = 1.202, *p* = 0.125) (Table 5).

### 3.3 Amoxapine (AXPN) outcomes

In this analysis of AXPN-exposed patients vs. general control patients, stratified by age, no statistically significant associations emerged for most infectious or inflammatory outcomes, though several results indicated noteworthy trends particularly among older adults (Table 6). COVID-19 incidence did not differ significantly between AXPN users and controls in any age group. The risk ratio was near unit across all strata: 0.933 in those aged 70–90 (*p* = 0.785), 1.158 in those aged 50–69 (*p* = 0.346), and 1.500 in the youngest cohort (*p* = 0.160). Likewise, no significant differences were seen in pneumonia, sepsis, or bronchitis/LRTIs. ENT/dental infections showed a consistent trend toward reduced incidence in AXPN users, particularly in the 70–90 age group (RR = 0.522, *p* = 0.058) and the 50–69 group (RR = 0.526, *p* = 0.089). No consistent pattern was observed for UTIs, with risk ratios varying by age group and no significant findings. Leukocytosis was significantly more common in AXPN users aged 70–90 (RR = 1.500, *p* = 0.033), but not in the other age groups. Elevated CRP and ESR levels were not significantly different from controls, though ESR trended higher in the 70–90 group (RR = 2.000, *p* = 0.063) but not statistically significant (Table 6).

**Table 6:** Association between AXPN exposure and infectious, inflammatory, and clinical outcomes in patients compared to a general control group (without baseline antidepressant or antipsychotic exposure).

Outcomes	Age 70–90 (*n* = 426)		Age 50–69 (*n* = 270)		Aged 18–49 (*n* = 247)	
	Risk ratio	*p*-value	Risk ratio	*p*-value	Risk ratio	*p*-value
*C. diff*	0.750 (0.36,1.57)	0.442	1.000 (0.42,2.36)	1.000	1.000 (0.42,2.36)	1.000
Pneumonia	1.529 (0.84,2.78)	0.159	1.000 (0.42,2.36)	1.000	1.000 (0.42,2.36)	1.000
Sepsis	1.182 (0.52,2.61)	0.679	–	–	1.000 (0.42,2.36)	1.000
COVID-19	0.933 (0.57,1.53)	0.785	1.158 (0.64,2.09)	0.346	1.500 (0.85,2.65)	0.160
TB	–	–	1.000 (0.42,2.36)	1.000	1.000 (0.42,2.36)	1.000
HSV	1.000 (0.42,2.38)	1.000	1.000 (0.42,2.36)	1.000	1.000 (0.42,2.36)	1.000
Bacteremia	1.000 (0.42,2.38)	1.000	–	–	1.000 (0.42,2.36)	1.000
Bronchitis/ LRTIs	0.765 (0.38,1.55)	0.457	1.000 (0.42,2.36)	1.000	1.000 (0.42,2.36)	1.000
UTIs	1.333 (0.85,2.10)	0.212	0.769 (0.34,1.72)	0.523	1.000 (0.42,2.36)	1.000
ENT/Dental infections	0.522 (0.26,1.04)	0.058	0.526 (0.25,1.11)	0.089	0.667 (0.38,1.18)	0.160
Inc. CRP	1.700 (0.79,3.67)	0.171	1.000 (0.42,2.36)	1.000	1.000 (0.42,2.36)	1.000
Inc. ESR	2.000 (0.95,4.22)	0.063	–	–	1.000 (0.42,2.36)	1.000
Inc. leukocytes	1.500 (1.03,2.19)	0.033*	0.950 (0.52,1.74)	0.868	1.308 (0.65,2.64)	0.451
Mortality	1.000 (0.42,2.38)	1.000	–	–	1.000 (0.42,2.36)	1.000
ICU admissions	–	–	–	–	–	–

*P*-values less than 0.05 are marked with an asterisk.

No differences were seen in HSV, TB, bacteremia, or mortality across any age group. ICU admission data were too sparse for analysis. Notably, most point estimates for rare infectious outcomes like HSV or TB remained fixed at 1.000 with wide confidence intervals, reflecting data sparsity and limited power in this subsample (Table 6).

The next analysis evaluated the effects of AXPN exposure vs. fluoxetine use across age groups (70–90, 50–69, and 18–49) on a range of infectious and inflammatory outcomes. As shown in Table 7, while most outcomes did not yield statistically significant differences, there were notable findings related to *C. difficile* infection and leukocytosis in the oldest cohort.

**Table 7:** Association between AXPN exposure and infectious, inflammatory, and clinical outcomes compared to patients taking fluoxetine.

Outcomes	Age 70–90 (*n* = 424)		Age 50–69 (*n* = 278)		Aged 18–49 (*n* = 252)	
	Risk ratio	*p*-value	Risk ratio	*p*-value	Risk ratio	*p*-value
*C. diff*	0.458 (0.23,0.92)	0.025*	0.667 (0.31,1.46)	0.306	1.000 (0.42,2.37)	1.000
Pneumonia	0.862 (0.52,1.45)	0.574	1.000 (0.42,2.37)	1.000	1.000 (0.42,2.37)	1.000
Sepsis	0.813 (0.40,1.67)	0.571	1.000 (0.42,2.37)	1.000	1.000 (0.42,2.36)	1.000
COVID-19	0.875 (0.54,1.43)	0.592	0.967 (0.60,1.57)	0.891	0.844 (0.52,1.37)	0.491
TB	–	–	–	–	1.000 (0.42,2.37)	1.000
HSV	1.000 (0.42,2.39)	1.000	–	–	1.000 (0.42,2.37)	1.000
Bacteremia	1.000 (0.42,2.39)	1.000	–	–	–	–
Bronchitis/ LRTIs	1.400 (0.63,3.12)	0.408	1.000 (0.42,2.36)	1.000	1.000 (0.42,2.37)	1.000
UTIs	0.975 (0.64,1.48)	0.906	1.000 (0.42,2.36)	1.000	1.000 (0.42,2.37)	1.000
ENT/Dental infections	1.083 (0.50,2.35)	0.840	1.000 (0.42,2.36)	1.000	0.846 (0.34,1.85)	0.678
Inc. CRP	0.810 (0.43,1.51)	0.507	1.000 (0.42,2.36)	1.000	1.000 (0.42,2.36)	1.000
Inc. ESR	–	–	1.000 (0.42,2.36)	1.000	1.000 (0.42,2.36)	1.000
Inc. leukocytes	0.685 (0.51,0.92)	0.012*	0.655 (0.38,1.14)	0.131	1.059 (0.56,2.01)	0.861
Mortality	1.000 (0.42,2.38)	1.000	–	–	–	–
ICU admissions	–	–	–	–	–	–

*P-values less than 0.05 are marked with an asterisk.

Among patients aged 70–90, AXPN use was associated with a significantly lower risk of *C. difficile* infection compared to fluoxetine use (RR = 0.458, 95% CI: 0.23–0.92, *p* = 0.025). This protective association was not observed in the 50–69 (*p* = 0.306) or 18–49 (*p* = 1.000) age groups. A statistically significant reduction in leukocytosis was also observed in the 70–90 age group (RR = 0.685, *p* = 0.012). This trend was not significant in the younger groups, with *p* = 0.131 for ages 50–69 and *p* = 0.861 for ages 18–49. No differences were detected for other inflammatory markers, including CRP and ESR, where risk ratios remained close to 1.000 with wide confidence intervals. For respiratory infections such as pneumonia, sepsis, and bronchitis/LRTIs, no significant differences were detected in any age stratum. Point estimates for these outcomes hovered near 1.000, with all *p*-values well above 0.05, which suggest equivalency in risk between AXPN and fluoxetine users.

The comparison between AXPN and fluoxetine in patients aged 70–90 revealed a trend toward improved survival free from *C. difficile* infection among AXPN users (Table 8). The survival probability was 96.8%, with HR of 0.523. While this difference did not reach conventional statistical significance (*log-rank p* = 0.082), the result is clinically suggestive and aligns with earlier findings of reduced *C. difficile* incidence in this age group (as seen in Table 7). The proportional hazards assumption was met (proportionality *p* = 0.654), supporting the validity of the model.

**Table 8:** Kaplan–Meier survival analysis for statistically significant infectious and laboratory outcomes by age-stratified group of patient comparisons of AXPN vs. control and fluoxetine.

Outcome	Group (Age range)	Survival probability	Log rank *p*-value	Hazard ratio	Proportionality *p*-value
*C. diff*	A vs. F (70–90)	96.804	0.082	0.523	0.654
Leukocytosis	A vs. C (70–90)	96.883	0.531	1.336	0.274
	A vs. F (70–90)	84.375	0.138	0.769	0.215

A = AXPN; C = General Control (without baseline antidepressant or antipsychotic exposure); F = Fluoxetine.

### 3.4 Sensitivity analysis

Sensitivity analyses using alternative follow-up windows demonstrated that the association between TFP exposure and reduced ENT/dental infection risk was highly consistent over time. Compared with both fluoxetine-treated and general-control cohorts, TFP users exhibited significantly lower rates of ENT/dental infections at 6, 12, and 24 months, with risk ratios ranging from 0.274 to 0.359 (all *p* < 0.0001) (Table 9). In contrast, the association between TFP exposure and COVID-19 risk appeared to be more time-dependent. Compared with fluoxetine-treated controls, no significant difference was observed at 6 months (RR 1.104, 95% CI 0.95–1.28; *p* = 0.198), whereas significantly lower risks emerged at 12 months (RR 0.850, 95% CI 0.74–0.98; *p* = 0.024) and became more pronounced at 24 months (RR 0.680, 95% CI 0.60–0.78; *p* < 0.0001). Similarly, compared with general controls, a significant reduction in COVID-19 risk was observed at 12 months (RR 0.407, 95% CI 0.24–0.69; *p* = 0.0005), although this association was not present at 6 months or 24 months. Overall, these findings suggest that the observed association between TFP and reduced ENT/dental infections was robust across multiple follow-up periods, whereas associations with COVID-19 varied depending on the duration of follow-up.

**Table 9:** Select expanded sensitivity analyses at 6-months, 12-months, and 24-months for COVID-19 and ENT/dental infection outcomes.

Outcome	Compar.	6 Month RR (95% CI)	*p*-value	12 Month RR (95% CI)	*p*-value	24 Month RR (95% CI)	*p*-value
COVID-19	TFP vs. Fluoxetine	1.104 (0.95, 1.28)	0.198	0.850 (0.74, 0.98)	0.024	0.680 (0.60, 0.78)	<0.0001
COVID-19	TFP vs. Control	0.729 (0.43,1.23)	0.233	0.407 (0.24, 0.69)	0.0005*	0.939 (0.82, 1.07)	0.345
ENT/Dental infections	TFP vs. Fluoxetine	0.359 (0.25, 0.52)	<0.0001	0.314 (0.23, 0.42)	<0.0001	0.306 (0.24, 0.39)	<0.0001
ENT/Dental infections	TFP vs. Control	0.347 (0.24, 0.50)	<0.0001	0.292 (0.22, 0.39)	<0.0001	0.274 (0.22, 0.35)	<0.0001

*P*-values less than 0.05 are considered significant.

As an exploratory secondary analysis, selected outcomes were directly compared between the TFP and AXPN cohorts. These outcomes were chosen because they represented the most clinically relevant and reproducible findings identified in the primary analyses and therefore provided the most informative basis for head-to-head comparison. Compared with AXPN, TFP exposure was associated with significantly lower risks of COVID-19 (RR 0.671, 95% CI 0.54–0.83; *p* = 0.003) and ENT/dental infections (RR 0.312, 95% CI 0.24–0.40; *p* < 0.0001). Conversely, TFP users demonstrated a higher incidence of leukocytosis than AXPN users (RR 1.532, 95% CI 1.27–1.85; *p* < 0.0001). No significant difference was observed between the cohorts for pneumonia (RR 1.101, 95% CI 0.88–1.38; *p* = 0.390) (Table 10).

**Table 10:** Select expanded sensitivity analyses comparing TFP and AXPN cohorts for COVID-19, ENT/dental infection, pneumonia, and leukocytosis outcomes.

Outcome	Risk Ratio	*p*-value
COVID-19	0.671 (0.54–0.83)	0.003
ENT/Dental infections	0.312 (0.24–0.40)	<0.0001
Pneumonia	1.101 (0.88–1.38)	0.390
Leukocytosis	1.532 (1.27–1.85)	<0.0001

*P*-values less than 0.05 are considered significant.

## 4 Discussion

This large retrospective cohort study investigated whether exposure to the TFP and AXPN was associated with altered risks of infectious and inflammatory outcomes in real-world clinical populations. Previous preclinical studies, including mouse models and human cell line experiments, suggested that these compounds may exert protective effects against infections through host-directed mechanisms (8, 9). Our study sought to determine whether similar associations could be observed at the population level using a large electronic health record database.

One of the most reproducible findings was the association between TFP exposure and increased leukocytosis across multiple analyses and age groups. Leukocytosis is a nonspecific finding that may occur in the setting of infection, systemic inflammation, stress, medication effects, or immune activation. While leukocytosis is often interpreted as a marker of infection or systemic inflammation, the clinical significance of the observed leukocytosis in our study remains uncertain. Leukocytes, particularly neutrophils, play critical roles in pathogen clearance, tissue remodeling, and recovery from infection-related injury (13). Preclinical studies have demonstrated that TFP can activate host-directed immune pathways, including IL-33–responsive neutrophil recruitment, enhanced autophagy, and improved bacterial clearance (4). However, these findings cannot establish that the leukocytosis observed in our clinical cohort represents beneficial immune activation or immune priming. Accordingly, this finding should be interpreted cautiously, and further prospective and mechanistic studies are needed.

Importantly, a similar leukocytosis signal was also observed among older AXPN-treated individuals when compared with fluoxetine and control cohorts, suggesting that modulation of innate immune responses may be present in both drug exposures within specific subgroups. However, the leukocyte signal in AXPN was less consistent across analyses and did not extend to the broader infection-related outcomes observed with TFP. This indicates that the immunomodulatory effects observed with TFP are more robust and widespread, whereas those associated with AXPN appear more limited and context-dependent. This interpretation is further supported by sensitivity analyses directly comparing TFP and AXPN, which demonstrated a significantly increased risk of leukocytosis in the TFP group (Table 10). The reproducibility of leukocytosis findings in older adults may reflect age-related immune dysregulation and increased sensitivity of innate immune pathways to pharmacologic modulation in this subgroup. Because excessive or dysregulated leukocytosis can also contribute to inflammatory pathology (14), further studies incorporating leukocyte subsets, longitudinal immune profiling, and mechanistic validation are required.

Another notable finding was the reduced incidence of ENT/dental infections among TFP users. TFP belongs to the phenothiazine class of compounds, which have demonstrated anti-mycobacterial activity as well as bactericidal effects against multiple organisms (15–17). TFP has been shown to promote autophagy, enhance bacterial clearance, and inhibit biofilm formation (18, 19). In contrast, AXPN did not demonstrate a comparable reduction in bacterial infection outcomes. This discrepancy suggests that the observed antimicrobial effects are not shared across all psychotropic agents and may instead be specific to phenothiazine derivatives such as TFP. TFP’s additional properties include functioning as bacterial efflux-pump inhibitors, particularly targeting the *Staphylococcus aureus* NorA system, thereby enhancing fluoroquinolone activity and reducing microbial persistence within biofilms (20). These mechanisms provide a biologically plausible explanation for the reduced rates of upper aerodigestive tract infections observed in TFP-treated patients. At the same time, the heterogeneous effects observed across different infectious outcomes suggest that TFP’s clinical impact is likely pathogen-specific and dependent upon the interplay between host immunity and microbial characteristics. Notably, these antimicrobial mechanisms have not been described for AXPN, supporting the interpretation that the observed effects are drug-specific rather than class-wide phenomena.

Trifluoperazine was also associated with reduced COVID-19 incidence compared with both general controls and fluoxetine-treated controls in age group 70–90. This observation is supported by several mechanistic studies demonstrating antiviral effects of TFP and related Functional Inhibitors of Acid SphingoMyelinAse (FIASMSA) compounds. TFP inhibits the acid sphingomyelinase/ceramide pathway required for SARS-CoV-2 host-cell entry, disrupting ceramide-enriched membrane platforms involved in viral fusion (21). TFP has also been shown to suppress viral replication through PERK-elf2α-mediated translational shutdown (22). Furthermore, epidemiologic studies of FIAMSA medications have reported lower COVID-19 hospitalization and mortality rates (23). Together, these findings provide a biologically plausible framework for the reduced COVID-19 incidence observed in our analysis. However, because the study period spanned multiple phases of the pandemic characterized by changing variants, vaccination rates, and testing availability, these results should be interpreted cautiously.

Although both TFP and AXPN demonstrated protective effects in preclinical infection models, the two drugs showed distinct patterns of association in the present study. TFP exhibited broader signals across infectious and inflammatory outcomes, whereas AXPN demonstrated more selective associations, most notably a reduced risk of *C. difficile* infection and leukocytosis among older adults compared to general controls and fluoxetine-treated controls. Previous work has shown that AXPN enhances autophagy and host defense responses against *C. difficile* and *Mycobacterium* species in the experimental models (2, 6, 7, 24). However, many of these protective effects were not consistently observed at the population level. This discrepancy may reflect the smaller AXPN cohort size, reduced statistical power, differences in underlying mechanisms, or the possibility that AXPN exerts more modest but critical immunomodulatory effects, as noted in animal models, than TFP. Unlike TFP, AXPN does not possess phenothiazine-associated antimicrobial properties or documented FIAMSA activity, potentially explaining the more limited range of associations observed in this study. In the future, it would be important to assess whether TFP/AXPN enhance tolerance to bacterial and viral antigens by promoting adaptive immunity. Further, whether these drugs could select antigen-specific protective or immunodominant but cross-reactive B cell clones to provide protection against infection needs to be addressed (25, 26). It will be intriguing in the future to also glean whether T cell immunity is altered in some way by these drugs.

## 5 Limitations

Although sensitivity analyses using alternative follow-up periods consistent with guideline-recommended treatment durations yielded similar results, the choice of a primary two-year outcome window may still influence effect estimates. Therefore, residual uncertainty remains regarding optimal temporal framing of exposure–outcome associations in real-world settings. Similarly, the 30-day window chosen for CRP and neutrophil assessments were designed to capture acute immune shifts following medication exposure. As expected, psychiatric diagnoses differed across cohorts because TFP, AXPN, and fluoxetine are prescribed for different clinical indications. While age, sex, and comorbidity adjustment were performed, residual confounding related to underlying psychiatric illness, lifestyle, healthcare utilization, and disease severity may have influenced observed associations. Medication exposure was determined from prescription records only; information regarding dose, treatment duration, cumulative exposure, and medication adherence was unavailable. Consequently, these findings should be considered exploratory and hypothesis-generating rather than confirmatory.

The large number of outcomes examined increases the risk of type I error. As this study was designed as an exploratory analysis, no correction for multiple comparisons was applied. Consequently, findings near the threshold of statistical significance should be interpreted as hypothesis-generating and require validation in independent cohorts. While matching for age and sex helped to reduce confounding, residual confounding remains possible.

## 6 Conclusion

Despite these limitations, our findings highlight the utility of real-world clinical data for identifying potential drug repurposing signals. TFP’s repeated association with reduced infection-related outcomes, supported by mechanistic literature, suggests potential therapeutic value as a host-directed agent. At the same time, the observed increases in leukocytosis and inflammatory markers underscore the complexity of its immunological profile and reinforce the need for pathogen- and context-specific interpretation.

The divergent effects observed between TFP and AXPN further emphasize that immunomodulatory properties are not uniform across psychotropic drug classes and may instead be compound-specific. Future work should include mechanistic immune assays, pathogen-specific analyses, and prospective validation studies to disentangle these effects. In parallel, preclinical studies in relevant animal models to dissect the host-innate pathways modulated by these drugs would shed light on mechanisms of bacterial clearance. Ultimately, this line of inquiry may contribute to the development of host-directed therapies capable of addressing both emerging viral threats and the global burden of antimicrobial resistance in bacterial pathogens.

## Data Availability

The original contributions presented in the study are included in the article/supplementary material, further inquiries can be directed to the corresponding author.
